# Supplementary material for: Antivirulence Agent as an Adjuvant of β-Lactam Antibiotics in Treating Staphylococcal Infections
Source: Antibiotics (Basel). 2022 Jun 17;11(6):819. doi: 10.3390/antibiotics11060819 (PMC9219823; doi:10.3390/antibiotics11060819)
Supplement: Supplementary file 1 [file antibiotics-11-00819-s001.zip › antibiotics-1746946-supplementary.pdf]

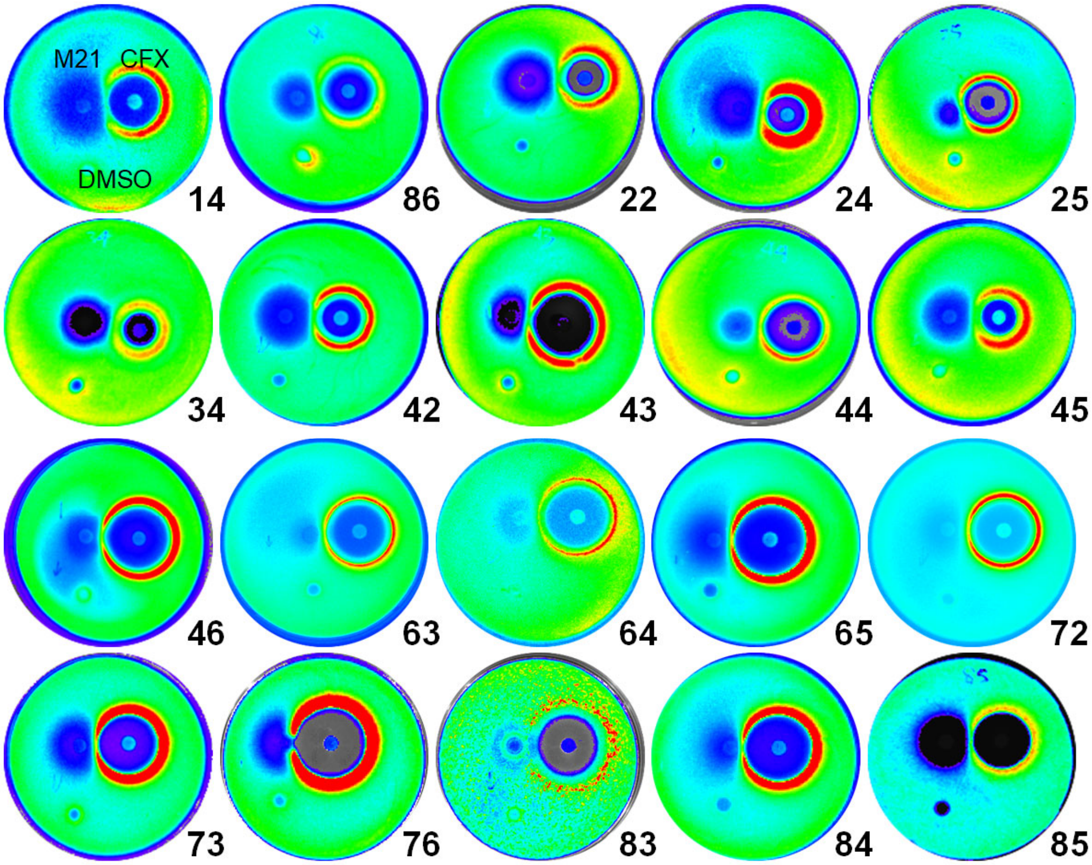

Figure S1. Paper disc assay showing the interaction of M21 with cefoxitin.

Cefoxitin (4mM, 5 µl) induced *hla* expression against different clinical isolates. M21 (4 mM, 5µl) repressed *hla* expression even in the induced state.
